# LncRNA *HOTAIR* impairs the prognosis of papillary thyroid cancer via regulating cellular malignancy and epigenetically suppressing *DLX1*

**DOI:** 10.1186/s12935-022-02817-2

**Published:** 2022-12-09

**Authors:** Feng-Chih Kuo, Yu-Ting Wang, Chia-Hsin Liu, Yao-Feng Li, Chieh-Hua Lu, Sheng-Chiang Su, Jhih-Syuan Liu, Peng-Fei Li, Chia-Luen Huang, Li-Ju Ho, Chien-Ming Lin, Chien-Hsing Lee

**Affiliations:** 1 Division of Endocrinology and Metabolism, Department of Internal Medicine, Tri-Service General Hospital, National Defense Medical Center, Taipei, Taiwan; 2 Department and Graduate Institute of Life Biochemistry, National Defense Medical Center, Taipei, Taiwan; 3 Division of Pulmonary and Critical Care Medicine, Department of Internal Medicine, Tri-Service General Hospital, National Defense Medical Center, Taipei, Taiwan; 4 Department of Pathology, Tri-Service General Hospital, National Defense Medical Center, Taipei, Taiwan; 5 Department of Pediatrics, Tri-Service General Hospital, National Defense Medical Center, Taipei, Taiwan

**Keywords:** lncRNA, *HOTAIR*, Papillary thyroid cancer, DLX1

## Abstract

**Purpose:**

Papillary thyroid cancer (PTC) is the most common endocrine malignancy with a fast-growing incidence in recent decades. *HOTAIR* as a long non-coding RNA has been shown to be highly expressed in papillary thyroid cancer tissues with only a limited understanding of its functional roles and downstream regulatory mechanisms in papillary thyroid cancer cells.

**Methods:**

We applied three thyroid cancer cell lines (MDA-T32, MDA-T41 and K1) to investigate the phenotypic influence after gain or loss of *HOTAIR*. The Cancer Genome Atlas (TCGA) database were utilised to select candidate genes possibly regulated by *HOTAIR* with validation in the cellular system and immunohistochemical (IHC) staining of PTC tissues.

**Results:**

We observed *HOTAIR* was highly expressed in MDA-T32 cells but presents significantly decreased levels in MDA-T41 and K1 cells. *HOTAIR* knockdown in MDA-T32 cells significantly suppressed proliferation, colony formation, migration with cell cycle retardation at G1 phase. On the contrary, *HOTAIR* overexpression in MDA-T41 cells dramatically enhanced proliferation, colony formation, migration with cell cycle driven toward S and G2/M phases. Similar phenotypic effects were also observed as overexpressing *HOTAIR* in K1 cells. To explore novel *HOTAIR* downstream mechanisms, we analyzed TCGA transcriptome in PTC tissues and found *DLX1* negatively correlated to *HOTAIR*, and its lower expression associated with reduced progression free survival. We further validated *DLX1* gene was epigenetically suppressed by *HOTAIR* via performing chromatin immunoprecipitation. Moreover, IHC staining shows a significantly stepwise decrease of DLX1 protein from normal thyroid tissues to stage III PTC tissues.

**Conclusions:**

Our study pointed out that *HOTAIR* is a key regulator of cellular malignancy and its epigenetic suppression on *DLX1* serves as a novel biomarker to evaluate the PTC disease progression.

**Supplementary Information:**

The online version contains supplementary material available at 10.1186/s12935-022-02817-2.

## Introduction

Thyroid cancer is the most common endocrine malignancy and presents a rapid growing annual incidence in recent decades. The increased incidence is probably due to early detection using high-resolution ultrasonography and commonly diagnosed at a younger age [1]. Histologically, there are five major types of thyroid cancer including papillary, follicular, poorly differentiated, anaplastic, and medullary thyroid cancer. Among them, papillary thyroid cancer (PTC) accounts for the majority (around 80%) of thyroid cancer [2]. The first-line treatment of PTC is total thyroidectomy, then followed with radioactive iodine ablation of thyroid remnant and thyroid hormone suppression of thyroid-stimulating hormone (TSH). Generally, the overall survival is more than 90% [3]. However, recent research disclosed there are still around 20% of PTC patients encountered disease recurrence with lymph nodes metastasis, causing the consequence of increased mortality [4]. Indeed, heterogeneity within the category of PTC has been reported and only could be roughly characterized by some histological features and molecular markers, such as *BRAF*^*V600E*^ mutation and *RET/PTC* rearrangements [5]. Besides, using transcriptional and mutational landscape, well-differentiated thyroid cancer could be classified as three molecular subtypes including *BRAF*-like, *RAS*-like, and Non-*BRAF*-Non-*RAS* [6]. Notably, increased prevalence of metastasis, gross extra-thyroidal extension, and multifocality were found in the *BRAF*-like subtype [7]. Therefore, further investigation on the genetic and epigenetic alternations of *BRAF*-like PTC to identify novel prognosis associated markers is of vital importance.

Long non-coding RNAs (lncRNAs) were defined as RNA transcripts with more than 200 nucleotides and not translated into proteins. Recently, many lncRNAs have been recognized to present dynamic expression during the mammalian organ development [8] and their dysregulations were observed among numerous cancers [9], indicating lncRNAs could function as important regulators during organogenesis and involve in carcinogenesis. *HOTAIR* (HOX Transcript Antisense Intergenic RNA), as one of the well-known lncRNAs, has recently been noted to possess potential roles in PTC. *HOTAIR* could be detected in serum and the levels might serve as a diagnostic value to distinguish thyroid benign nodule and PTC [10]. Also, PTC patients with lymph node metastasis have significantly higher serum *HOTAIR* levels than them without metastasis [11]. Moreover, PTC tissues were shown to express increased *HOTAIR* levels, which further positively correlated with advanced pathological stages and poor prognosis of patients [12]. These results point out *HOTAIR* likely plays functional roles during PTC carcinogenesis and metastasis and can serve as a potential marker for evaluating disease status of PTC patients.

To explore *HOTAIR* mediated molecular mechanisms in thyroid cancer, several groups have examined the *HOTAIR*-miRNA-mRNA competitive endogenous RNA networks in multiple thyroid cancer cell lines. For examples, using papillary (TPC-1) and follicular (FTC-133) thyroid cancer cell lines, *HOTAIR* was showed to sponge miR-1, activate *CCND2* expression, and promote thyroid cancer progression [13]. Also, *HOTAIR* can down-regulate miR-488-5p with upregulation of NUP205 and Bcl-2 to enhance growth, migration, and invasion of the papillary (BCPAP) thyroid cancer cell [14]. Similarly, *HOTAIR* promotes cell viability, migration, and invasion in papillary (TPC-1) and follicular (FTC-133) thyroid cancer cells via counter-regulating miR-17-5p [15]. Using papillary (BCPAP) and anaplastic (HTh-7 and CAL-62) thyroid cancer cells, *HOTAIR*/miR-761 sponge can regulate PPME1 to promote cell proliferation and inhibit cell apoptosis [16]. Therefore, it is undoubtedly that *HOTAIR* can sponge various miRNAs to enhance cell malignant behaviours in thyroid cancer cells.

However, *HOTAIR* mediated epigenetic mechanisms are not limited to miRNAs sponge. *HOTAIR* can also silence genes *in trans* via interacting with polycomb repressive complex 2 (PRC2) at the 5’ end and lysine-specific histone demethylase 1 (LSD1) at the 3’ end to modulate H3K27 trimethylation and H3K4 demethylation, respectively [17]. To our knowledge, this mechanism in papillary thyroid cancer has not been investigated before. Additionally, previous research mainly used the traditional PTC cell lines, such as BCPAP and TPC-1. Therefore, in this study, we utilized two newly characterized PTC cell lines (MDA-T32 and MDA-T41) [18] to evaluate the *HOTAIR* phenotypic effects on cellular biology after gain or loss of its expression. The Cancer Genome Atlas (TCGA) database were used to select genes that potentially repressed by *HOTAIR* in PTC tissues. The cellular models, chromatin immunoprecipitation assay and immunohistochemical (IHC) staining of PTC tissues were further applied to validate the putative *HOTAIR-*suppressed gene and evaluate its association with the clinical staging of PTC. We aim to strengthen current understandings of *HOTAIR*-mediated carcinogenesis and epigenetic alternations in PTC with identification of novel prognosis associated markers for clinical application.

## Results

### HOTAIR was overexpressed in PTC, associated with decreased overall survival, and variably expressed among four PTC cell lines

**Fig. 1:**
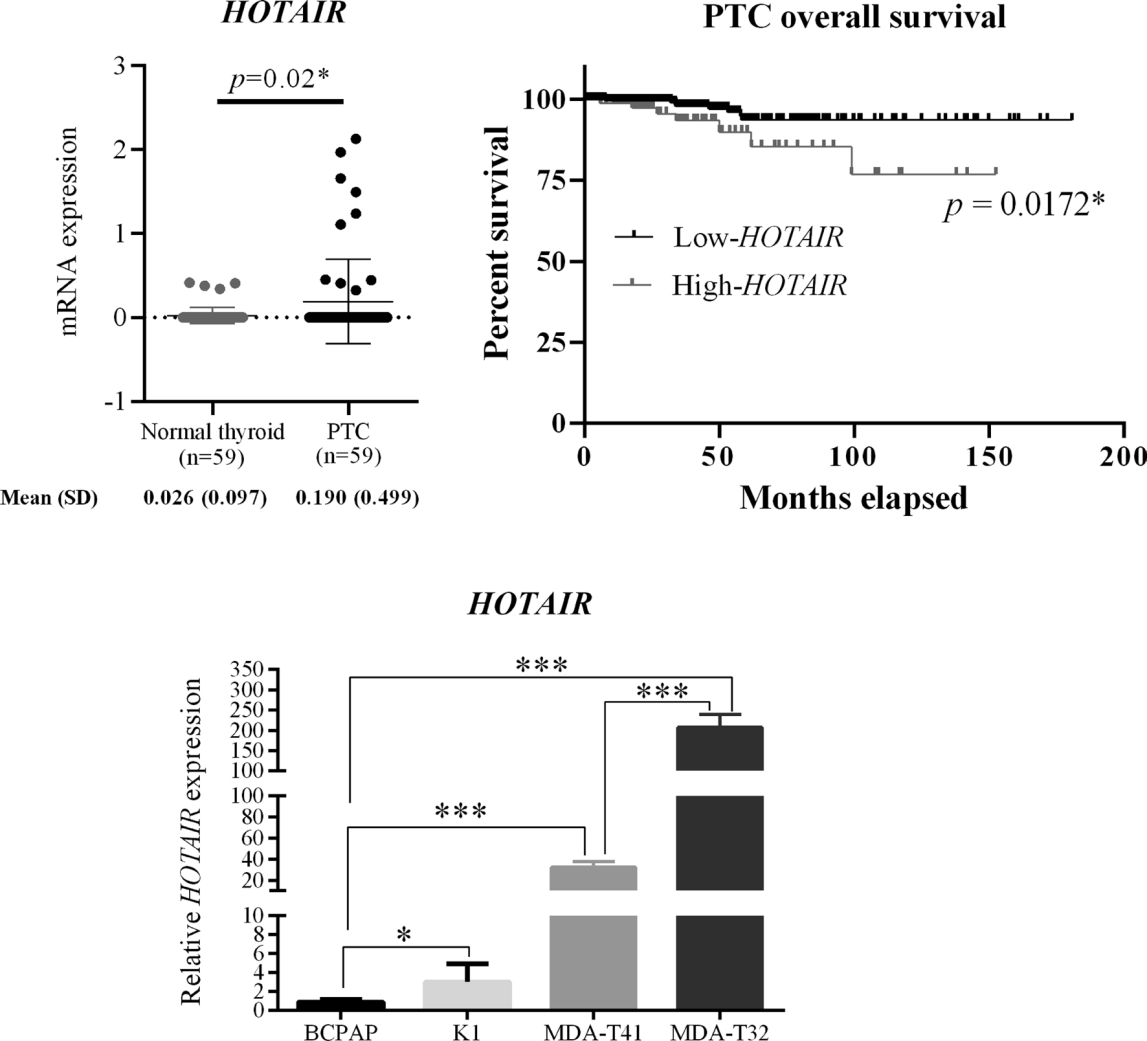
*HOTAIR* was variably overexpressed in PTC tissues and cell lines and associated with survival probability. **A** *HOTAIR* expression in PTC tissues and corresponding adjacent normal thyroid tissues were obtained from TCGA database and specifically compared. **B** Kaplan–Meier survival analysis revealed patients with high *HOTAIR* expression (n = 96) have decreased overall survival comparing to ones with low *HOTAIR* levels (n = 417). Cut off value: 0.4078 **C** Relative *HOTAIR* expression was measured by real-time qPCR and compared between four PTC cell lines (n = 3). Data are presented as means ± SD. Wilcoxon Signed Ranks test was used to compare *HOTAIR* levels between PTC tissues and adjacent normal thyroid tissues. Independent Student T-test for comparing *HOTAIR* levels among PTC cell lines. Log-rank test was used to compare survival probability. *p < 0.05; **p < 0.01; ***p < 0.001

Using the TCGA database, we compare the gene expression between PTC tissues and their matched adjacent normal thyroid tissues (n = 59) and the PTC tissues were shown to present significantly increased *HOTAIR* levels (Fig. 1A). Besides, the elevated *HOTAIR* expression was further associated with reduced survival probability in PTC patients (Fig. 1B), suggesting *HOTAIR* might play functional roles to steer the tumorigenesis and disease progression of PTC. Previous studies have found the BCPAP cells express significantly higher *HOTAIR* levels than the normal thyroid follicular cells [14, 15]. Here, we further compared *HOTAIR* expression among four PTC cells (BCPAP, K1, MDA-T41 and MDA-T32). Notably, although these PTC cell lines all carried *BRAF*^*V600E*^ mutation, but they expressed very differential *HOTAIR* levels (Fig. 1C), implying *HOTAIR* might be a *BRAF*^*V600E*^ mutation independent epigenetic regulator to alter the malignant behaviours of PTC cells. Using the TCGA database, the *HOTAIR* levels among the PTCs with different genetic background including *BRAF*^*V600E*^, *NRAS*, *HRAS* mutations and *RET* fusion were further compared (Additional file 1: Figure S1). Although the results showed no significant difference, but the PTCs with *BRAF*^*V600E*^ mutation do have high individual variation on *HOTAIR* expression, which is in line with our findings that there are differential *HOTAIR* levels among four PTC cell lines with *BRAF*^*V600E*^ mutation.

### Modulation of HOTAIR expression in two well-characterized PTC cells (MDA-T41 and MDA-T32) regulates cell proliferation, colony formation and cell migration

**Fig. 2:**
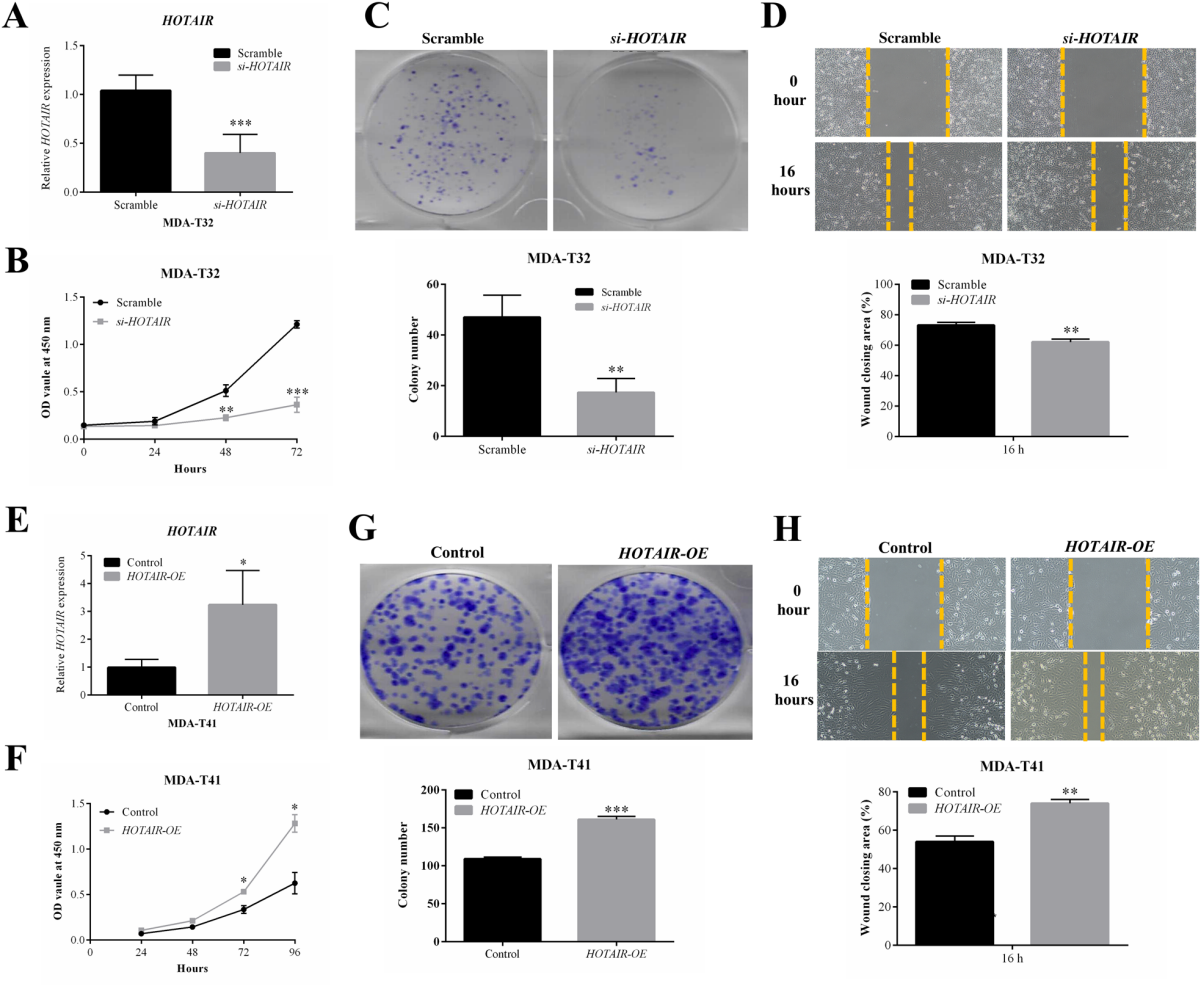
Modulation of *HOTAIR* expression in PTC cells regulates proliferation, colony formation and migration. **A** *HOTAIR* knockdown in the MDA-T32 cell line (*si-HOTAIR* cell) was achieved via adding *HOTAIR* siRNA and scramble siRNA was used to generate the Scramble cell (n = 3). **B, C** Cell proliferation rate of Scramble and *si-HOTAIR* cells was assessed by CCK-8 and colony formation assay (n = 3). **D** Cell migration of Scramble and *si-HOTAIR* cells was evaluated using wound-healing assay (n = 3). **E** Constitutive *HOTAIR* overexpression in the MDA-T41 cell line (*HOTAIR-OE* cell) was generated via lentiviral transduction and empty vector was utilized to generate the Control cell (n = 3). **F, G** Cell proliferation rate of Control and *HOTAIR-OE* cells was assessed by CCK-8 and colony formation assay (n = 3). **H** Cell migration of Control and *HOTAIR-OE* cells was evaluated using wound-healing assay (n = 3). Data are presented as means ± SD. Statistical significance was assessed using independent Student T-test. *p < 0.05; **p < 0.01; ***p < 0.001

Since MDA-T32 and MDA-T41 are the newly developed and well-characterized PTC cells [18] with differential *HOTAIR* expression (MDA-T32 presents highest *HOTAIR* expression with 6.4-fold higher levels than that in MDA-T41) (Fig. 1C), we applied MDA-T32 and MDA-T41 cell lines to perform *HOTAIR* knockdown and constitutive overexpression experiments, respectively. As shown in Fig. 2A and E, we successfully knocked down *HOTAIR* transcript in MDA-T32 and induced *HOTAIR* overexpression in MDA-T41. Then, *HOTAIR* knockdown in MDA-T32 substantially suppressed the cell proliferation rate (Fig. 2B), colony formation (Fig. 2C) and cell migration (Fig. 2D). In contrast, constitutive overexpression of *HOTAIR* in MDA-T41 remarkably increased the cell proliferation rate (Fig. 2F), colony formation (Fig. 2G) and cell migration (Fig. 2H). These results provide supportive evidence that *HOTAIR* as a lncRNA plays active roles to promote the cell malignant behaviours in PTC.

### Modulation of HOTAIR in PTC cell lines alters cell cycle phases and PTEN/p-AKT pathway

**Fig. 3:**
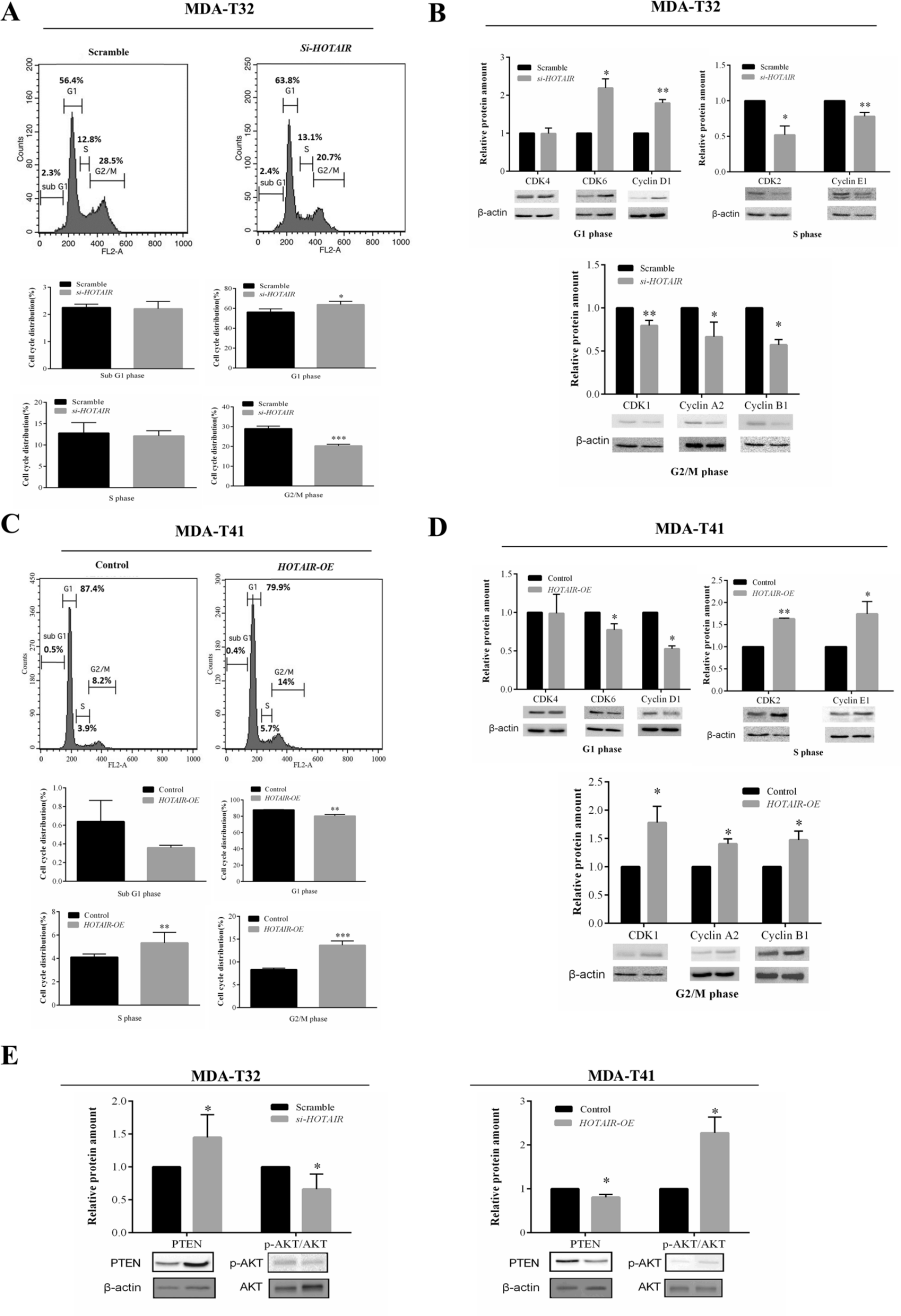
Modulation of *HOTAIR* expression in PTC cells regulates cell cycle. **A** Flow cytometry was performed among Scramble and *si-HOTAIR* cells to evaluate *HOTAIR* knockdown-mediated effects on cell cycle distribution (n = 3). **B** Protein markers of different cell phases were blotted on Scramble and *si-HOTAIR* cells (n = 3). **C** Flow cytometry was performed among Control and *HOTAIR-OE* cells to evaluate *HOTAIR* overexpression-mediated effects on cell cycle distribution (n = 3). **D** Protein markers of different cell phases were blotted on Control and *HOTAIR-OE* cells (n = 3). **E** PTEN and p-AKT/AKT ratio were blotted on Scramble/*si-HOTAIR* and Control/*HOTAIR-OE* cells to quantify their relative protein levels after regulating *HOTAIR* expression (n = 3). Data are presented as means ± SD. Statistical significance was assessed using independent Student T-test. *p < 0.05; **p < 0.01; ***p < 0.001

*HOTAIR* influences the cell proliferation rate and colony formation in PTC cell lines might base on its effects on cell cycle phases. Therefore, we further examine its effects on cell cycle phases via running flow cytometry and blotting the corresponding proteins. Significantly, *HOTAIR* knockdown in MDA-T32 cells retarded the cell cycle in the G1 phase with decreased ratio of G2/M phase distribution (Fig. 3A). Compatibly, in the MDA-T32 *si-HOTAIR* cells, G1 phase related proteins (CDK6 and Cyclin D1) were significantly increased, while S phase related proteins (CDK2 and Cyclin E1) and G2/M phase related proteins (CDK1, Cyclin A2 and Cyclin B1) were inhibited (Fig. 3B). On the contrary, *HOTAIR* overexpression in MDA-T41 cells shifted the cell cycle toward S and G2/M phases with decreased ratio of G1 phase distribution (Fig. 3C). Also, in the MDA-T41 *HOTAIR-OE* cells, S phase related proteins (CDK2 and Cyclin E1) and G2/M phase related proteins (CDK1, Cyclin A2 and Cyclin B1) were considerably enhanced, while G1 phase related proteins (CDK6 and Cyclin D1) were suppressed (Fig. 3D). To further strengthen these findings, we also overexpressed *HOTAIR* transcript in another low *HOTAIR*-expressed PTC cell line (K1). Again, we are able to observe that cell proliferation and colony formation were significantly enhanced, and cell cycle was shifted toward G2/M phase after *HOTAIR* overexpression (Additional file 1: Figure S2). Whereas the sub G1 phase was also significantly increased in the *HOTAIR*-overexpressed K1 cells. Considering the sub G1 phase generally represented the apoptotic cells [19], this phenomenon might be due to the insufficient nutrients to meet the demands during rapid cell proliferation [20].

Numerous studies have shown PTEN/p-AKT pathway heavily involved in regulating all phases of the cell cycle including G1/S [21] and G2/M transitions [22]. *PTEN* has also been shown to be epigenetically suppressed by *HOTAIR* [23]. Therefore, we blotted the PTEN and p-AKT in our *HOTAIR* knockdown and overexpression cellular system. Interestingly, PTEN/p-AKT pathway presented substantially corresponding changes after gain or loss of *HOTAIR* in PTC cell lines*. HOTAIR* knockdown in MDA-T32 enhanced PTEN production with decrease of p-AKT/AKT ratio, whereas *HOTAIR* overexpression in MDA-T41 suppressed PTEN amounts and remarkably increased the p-AKT/AKT ratio (Fig. 3E). These results indicate *HOTAIR* as a lncRNA can alter PTC cell cycle phases likely through regulating PTEN/p-AKT pathway.

### DLX1 was epigenetically suppressed by HOTAIR, and lower DLX1 levels in PTC tissues were associated with disease progression

**Fig. 4:**
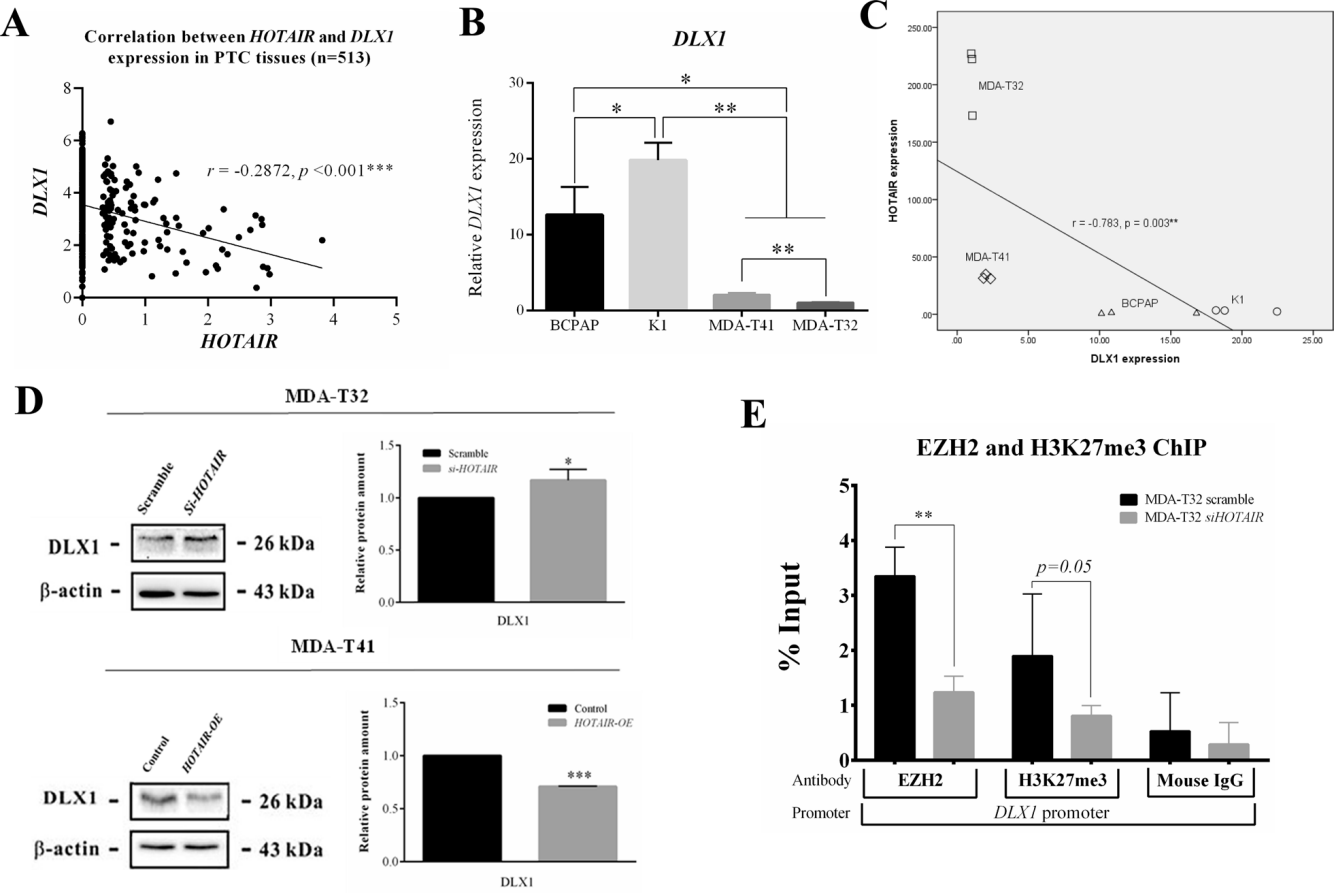
*DLX1* was negatively correlated with *HOTAIR* and epigenetically regulated by *HOTAIR.* **A** Correlation between *DLX1* and *HOTAIR* expression in PTC tissues was plotted and analyzed with Pearson’s correlation. **B** Relative *DLX1* expression was measured by real-time qPCR and compared between four PTC cell lines (n = 3). **C** Correlation between *HOTAIR* and *DLX1* expression was assessed among four PTC cell lines (n = 3). **D** DLX1 was blotted on Scramble/*si-HOTAIR* and Control/*HOTAIR-OE* cells to quantify its relative protein levels after regulating *HOTAIR* expression (n = 3). **E** EZH2 (n = 5) and H3K27me3 (n = 3) chromatin immunoprecipitation were performed on Scramble and *si-HOTAIR* cells. Data are presented as means ± SD. Statistical significance was assessed using paired or independent Student T-test; Mann–Whitney U test to examine the differential %Input between two cells, *p < 0.05; **p < 0.01; ***p < 0.001

**Fig. 5:**
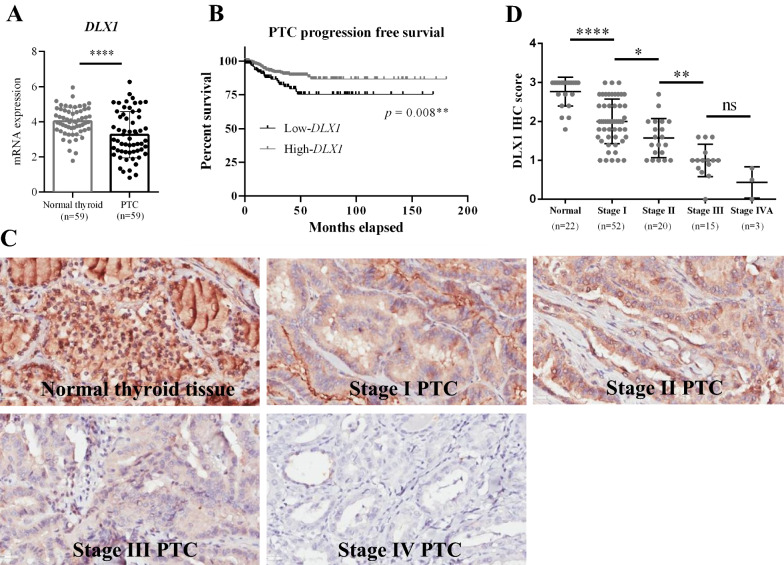
*DLX1* was associated with disease progression and immunohistochemical staining showed a stepwise decrease of DXL1 from normal thyroid tissues to advanced stages of PTC tissues. **A** **DLX1** expression in PTC tissues and corresponding adjacent normal thyroid tissues were obtained from TCGA database and specifically compared. **B** Kaplan–Meier survival analysis revealed patients with low *DXL1* expression (n = 129) have decreased progression free survival comparing to ones with high *DXL1* levels (n = 386). Cut off value: 2.5943. **C** Immunohistochemical staining (IHC) of DXL1 protein was performed in normal thyroid tissues and different stages of PTC tissues **D** IHC scores were calculated by staining intensity*staining percentage and compared between normal thyroid and different stages of PTC tissues. Data are presented as means ± SD. Statistical significance was assessed using paired Student T-test or one-way ANOVA post-hoc Tukey’s multiple comparisons test, *p < 0.05; **p < 0.01; ***p < 0.001

*HOTAIR* mediated recruitment of PRC2 complex, containing enhancer of zeste homolog 2 (EZH2) as a histone-lysine N-methyltransferase enzyme can increase H3K27 trimethylation to silence downstream genes *in trans* [17]. However, this mechanism has not been explored in papillary thyroid cancer till now. Therefore, we used the TCGA transcriptomic data of PTC tissues to select top 5 genes that were negatively correlated to *HOTAIR* expression (Additional file 1: Table S1). Then, we run real-time qPCR of these 5 genes in our *HOTAIR* knockdown and overexpression PTC cellular system. Notably, *DLX1* expression showed bidirectional corresponding changes after gain or loss of *HOTAIR* in PTC cell lines (Additional file 1: Figure S3), suggesting *DLX1* might be epigenetically suppressed by *HOTAIR*. Then, we plotted the expression of *DLX1* and *HOTAIR* in 513 PTC tissues, showing *DLX1* is significantly negatively correlated to *HOTAIR* (*r* = −0.2872, *p* < 0.001) (Fig. 4A). Besides, we also observed the highest *HOTAIR*-expressed PTC cell line (MDA-T32) expressed the lowest *DLX1* level (Fig. 4B) and the endogenous *DLX1* expression was negatively correlated with *HOTAIR* levels among four PTC cell lines (Fig. 4C). To further confirm *DLX1* as a *HOTAIR* epigenetically suppressed gene, we blotted the DLX1 protein in our *HOTAIR* knockdown and overexpression cellular system of PTC cell lines. In line with the results of real-time qPCR, DLX1 proteins were significantly increased in MDA-T32 *si-HOTAIR* cells and steadily suppressed in MDA-T41 *HOTAIR-OE* cells (Fig. 4D). Then, we performed chromatin immunoprecipitations on MDA-T32 scramble and *si-HOTAIR* cells and our results demonstrated the occupancies of EZH2 and H3K27me3 on *DLX1* promoter were meaningfully suppressed (*p* < 0.01 for EZH2 and *p* = 0.05 for H3K27me3) after *HOTAIR* knockdown (Fig. 4E). To examine the biological importance of DLX1 in PTC, *DLX1* expression between PTC tissues and matched adjacent normal thyroid tissues (n = 59) was compared and its association with disease prognosis was analyzed using TCGA database. Markedly, PTC tissues expressed significantly decreased *DLX1* levels (Fig. 5A) and the lower *DLX1* expression was associated with poor progression free survival in PTC (Fig. 5B). To prove this association in vivo, we performed DLX1 IHC staining among normal thyroid tissue and different stages of PTC tissues. Indeed, IHC staining shows a significantly stepwise decrease of DLX1 protein from normal thyroid tissues to stage III PTC tissues (Fig. 5C and D). Overall, our data disclosed *DLX1* was epigenetically repressed by *HOTAIR* and loss of DLX1 associates with advanced PTC stages. DLX1 will likely be a novel marker to evaluate the disease prognosis of PTC.

## Discussion

Although patients with PTC generally have good overall survival rate under standard therapy, but there is still a concern for the high disease recurrence, which could reach up to 20% during the long-term follow-up [4]. Also, there is still a difficulty to determine the optimal initial operational choice and postoperative therapeutic strategies (such as if radioiodine ablation is required post total thyroidectomy) for PTC patients. For examples, patients with 1–4 cm intrathyroidal PTC without evidence of contralateral lobe or cervical lymph nodes metastasis can receive either solely thyroid lobectomy or total thyroidectomy with/without radioiodine therapy [24]. One important issue is that the heterogenicity within the histological features and genetic/epigenetic signatures of PTC have not been thoroughly understood. Therefore, there is a vital importance to recognize or discover novel prognosis-associated markers of PTC patients for clinical evaluation.

Recently, lncRNA *HOTAIR* has been recognized as a practicable biomarker in patients with PTC. The *HOTAIR* expression in serum or in thyroid tissues collected via fine-needle aspiration biopsies can serve as one of useful markers to differentiate benign nodules and thyroid cancers [10, 25]. *HOTAIR* expression was shown to be significantly increased in PTC tissues, positively correlated with the advanced PTC stages, and negatively associated with overall survival probability of PTC patients [12]. In vitro experiments using PTC cell lines further demonstrate *HOTAIR* could promote cell proliferation, migration, and invasion via competitively sponging numerous microRNAs including miR-1 [13], miR-488-5p [14], miR-17-5p [15] and miR-761 [16]. In the present study, we observed similar findings that *HOTAIR* was highly expressed in PTC tissues and its higher expression associated with lower overall survival. Notably, we further found there are differential *HOTAIR* expression among four PTC cell lines (BCPAP, K1, MDA-T41 and MDA-T32), supporting there is indeed heterogenicity within the category of PTC. We further modulated *HOTAIR* expression in two newly developed and well-characterized PTC cell lines. We performed *HOTAIR* knockdown in high *HOTAIR*-expressed MDA-T32 and conducted *HOTAIR* overexpression in MDA-T41, which presented significantly lower *HOTAIR* levels compared to MDA-T32. In line with previous reports, we demonstrated *HOTAIR* can foster cell proliferation, colony formation and migration, redistribute cell cycle phases toward S phase (DNA replication) and G2/M phase (cell mitosis) and correspondingly change the signaling of PTEN/p-AKT pathway. Overall, *HOTAIR* is thought as an important epigenetic regulator that vigorously participates in carcinogenesis, tumor growth, progression, and metastasis of PTC.

Currently, there is still limited understanding regarding the upstream regulatory mechanisms for *HOTAIR* transcription. *HOTAIR* can be upregulated by estradiol in vitro via enriching the bindings of estrogen receptors (ERs) to its promoter [26] and genetic variants such as single nucleotide polymorphisms (SNPs) can also potentially influence the *HOTAIR* expression with tissue-specific pattern. Indeed, *Zhu *et al*.* revealed *HOTAIR* rs920778 T allele as a functional genetic variant associated with higher *HOTAIR* levels in both normal thyroid and PTC tissues [27]. *HOTAIR* expression could also be induced by the tumor microenvironment such as hypoxia [28]. Therefore, the intrinsic factor (genetic variation) and extrinsic factors (estradiol and hypoxia) might coordinate together to modulate *HOTAIR* expression and contribute PTC tumorigenesis and will require future investigation to clarify in depth.

To extend *HOTAIR* mediated epigenetic alterations in PTC beyond microRNAs sponge, we examine several genes presented negative correlation with *HOTAIR* expression in PTC tissues. Then, our attention was centred on the *DLX1* gene since it retained negative corresponding changes after gain or loss of *HOTAIR* in our PTC cellular system. We further identified *DLX1* gene was epigenetically repressed by *HOTAIR* via recruitments of PRC2 (EZH2) and H3K27me3 on its promoter region. Moreover, using the TCGA dataset and IHC staining of PTC tissues, we disclosed that *DLX1* expression was significantly reduced in PTC tissues and decrease of DLX1 was associated with advanced PTC stages and lowered progression free survival. These results indicate DLX1 as a novel marker with a promising application for predicting the disease prognosis of PTC patients.

*DLX1* belongs to the *DLX* homeobox family genes that encode transcription factors possessing critical roles in the morphogenesis of craniofacial structures, pharyngeal arches, forebrain, and sensory organs [29]. Particularly, pharyngeal arches are closely linked to the development of thyroid gland. The thyroid originates from a diverticular outgrowth of the primitive pharynx between the first and second pharyngeal arches [30] and the developmental genes such as *DLX* and *HOX* genes are both important for patterning the anterior/posterior and dorsal/ventral axes of the pharyngeal arches [31]. *HOX* genes are the most well-known subset of homeobox genes containing four chromosome clusters (*HOXA*, *HOXB*, *HOXC* and *HOXD*), which involved in the formation of various body structures during embryonic development [32]. *HOTAIR* is transcribed from the antisense strand of *HOX*C cluster in chromosome 12 and has been demonstrated to repress the expression of *HOXD* genes in chromosome 2 [17]. In this study, we further identified *DLX1* gene was epigenetically supressed by *HOTAIR*. Intriguingly, *DLX1* gene just locates next to the *HOXD* gene cluster, indicating the *HOTAIR* mediated histone modifications can inhibit several genes in the near position of the same chromosome. Based on RNA sequencing data of GTEx (Genotype-Tissue Expression) Analysis Release V8 (dbGaP Accession phs000424.v8.p2), thyroid tissue is listed as the 2nd rank tissue with high *DLX1* expression (only below to the brain tissues), whereas *HOTAIR* was lowly expressed in thyroid tissue (only above to the whole blood). Therefore, maintenance of low *HOTAIR* expression and high *DLX1* levels in the thyroid gland is essential for its normal development and functionality. In this paper, we further demonstrated their dysregulation can promote the carcinogenesis and disease progression of PTC. However, future investigation is still warranted to ascertain the functional roles of DLX1 in PTC.

In summary, *HOTAIR* is an important epigenetic regulator for steering the tumorigenesis and disease progression of PTC through modulating the cellular malignancy. We validated *HOTAIR* can modulate histone modifications to inhibit *DLX1* expression and loss of DLX1 was associated with advanced PTC stages and decreased progression free survival. Our results broaden *HOTAIR* mediated epigenetic regulatory networks in PTC to include histone modifications. Moreover, DLX1 was recognized as a novel prognosis associated biomarker in PTC likely to be practicable for clinical utilization.

## Materials and methods

### Analyze gene expression in PTC tissues and its association with clinical prognosis

The Cancer Genome Atlas (TCGA) thyroid cancer data were obtained from UCSC Xena (http://xena.ucsc.edu). A total of 59 PTC tissues and matched adjacent normal thyroid tissues were applied to compare the *HOTAIR* and *DLX1* expression. In survival analysis, *HOTAIR* and *DLX1* expression were obtained from 513 and 515 PTC tissues, respectively, and stratified as high or low based on the expression level with significant differences in the survival outcomes and the lowest log-rank *P*-value among subgroups using *Cutoff Finder* [33]. *HOTAIR* expression in 308 TCGA PTC tissues with different genetic alternations including *BRAF*^*V600E*^, *NRAS*, *HRAS* mutations and *RET* fusion were further compared. We also utilized the TCGA data to select the putative *HOTAIR* suppressed genes that present a significantly negative correlation with *HOTAIR* expression. The correlation between *HOTAIR* and *DLX1* expression (n = 513) was further plotted and analyzed by Pearson correlation.

### Cell lines and culture conditions

The human papillary thyroid cancer cell lines including BCPAP, K1, MDA-T41 and MDA-T32 were cultured in Roswell Park Memorial Institute (RPMI) 1640 medium with 10% fetal bovine serum (FBS) and 1% penicillin-streptomycin-amphotericin B at 37 ℃ in 5% CO_2_.

### HOTAIR knockdown using small interfering (si)RNA transfection

The *HOTAIR* siRNAs (Cat#N-187951-02, GE Healthcare Dharmacon) and scramble siRNA (Cat#D-001210-01, GE Healthcare Dharmacon) were used to generate the *HOTAIR* knockdown cells (MDA-T32 *si-HOTAIR*) and scramble control cells (MDA-T32 Scramble). 10 nM siRNAs with transfection reagent were added to the cells following the protocols supplied by the manufacturer. Further assays or experiments were performed at 48 h post-transfection.

### Generation of constitutive HOTAIR overexpression

The sequence of *HOTAIR* (NR_003716) was cloned from the LZRS-*HOTAIR* plasmid (Addgene plasmid #26110) and inserted into the pEGFP-Lv105 vector (Capital Biosciences) as the working *HOTAIR* plasmid. pEGFP-Lv105 empty vector was used as a control. Then, we applied lentiviral packaging kit (Cat#3D5F03, Origene) in HEK293T cells to produce lentiviral particles. The MDA-T41 cells were transduced with *HOTAIR* plasmid- or empty vector-lentiviral particles in 8 µg/ml hexadimethrine bromide (Sigma)-containing growth medium to generate MDA-T41 *HOTAIR-OE* cells or Control cells, respectively. Then, cells were revived in normal growth medium for an additional 24–28 h with subsequent selection in puromycin-containing growth medium.

### Cell proliferation assay

The Cell Counting Kit-8 (CCK-8) assay purchased from Dojindo Laboratories (Cat#TJ557, Kumamoto) was used to evaluate cell proliferation rate. All the experimental protocols were performed as described in the manufacturer’s instructions. Briefly, 1 × 10^3^ cells per well were seeded into a 96‑well plate and cultured at 37 °C. At the indicated time points, CCK‑8 solution was added to each well with 1:9 ratio (CCK-8: media) and cells were incubated at 37 °C for a further 3 h. The absorbance at 450 nm was measured with a microplate reader (The Synergy^™^ HT, Bio-Tek, Taiwan).

### Colony-forming unit assay

1 × 10^3^ cells per well were seeded in a 6-well plate for 7–14 days at 37 ℃ in 5% CO2. The cells were fixed with 4% formalin for 30 min, washed by PBS and stained with 0.1% crystal violet (Cat#MKCH6258, Sigma). The colony forming units were photographed and counted using Image J.

### Wound-healing assay

5 × 10^3^ cells per well were seeded in a 12-well plate to reach 80–90% confluence. Then, a small wound was created in each well with a gentle wash to remove cell fragments. Cells were cultured at 37 ℃ in 5% CO_2_ and images were taken under a microscope at 0 and 16 h.

### Flow cytometric analysis of the cell cycle

Briefly, cells with the culture medium were harvested, washed with PBS following 1300 rpm spinning for 5 min at 4 ℃ × 3 times, fixed with ice cold ethanol (75%), washed with 1% FBS contained PBS following 1300 rpm spinning for 5 min at 4 ℃ twice, then stained with propidium iodide (PI) solution containing 50 μg/ml PI (Sigma) and 10 μg/ml RNase A (Thermo Fisher Science). Images of the cell cycle were obtained by the FACSCalibur system (BD Biosciences).

### Gene expression analysis

Total RNA was extracted from cells using TRIzol reagent and cDNA synthesis was performed using a high-capacity cDNA reverse transcription kit (Cat#00984365, Thermo Fisher Science). SensiFAST SYBR No-ROX mix (Cat#98005, Bioline) was used for running quantitative real-time PCR in a LightCycler 480 (Roche). The ΔCT values of target genes were normalized to the ΔCt of stably expressed reference transcripts (*GAPDH*). The primer sequences used are listed in Additional file 1: Table S2.

### Protein extraction and western blot

Total cells are washed with ice-cold PBS and whole cell extracts were prepared using protein lysis buffer. The Pierce^™^ bicinchoninic acid (BCA) protein assay kit (Thermo Fisher Science) was used to quantify protein amount of the samples. All immunoblots are standardized to the same amounts of proteins per well. Then, proteins were separated by SDS-PAGE and transferred onto polyvinylidene difluoride (PVDF) membranes. Western blotting analysis was subsequently performed via incubating the membranes with primary antibodies against to proteins of interest under appropriate dilution. Then, horseradish peroxidase-conjugated secondary antibodies and the enhanced chemiluminescence assay were applied to visualize the signal. Band intensities are determined via using an UVP GelStudio^™^ PLUS Imager (Analytik Jena, Germany). The primary antibodies against CDK1 (1:1000, Cat#9116), CDK2 (1:1000, Cat#2546), CDK4 (1:1000, Cat#12790), CDK6 (1:1000, Cat#13331), Cyclin A2 (1:1000, Cat#91500), Cyclin B1 (1:1000, Cat#4135), Cyclin D1 (1:1000, Cat#55688), Cyclin E1 (1:1000, Cat#4129), p-AKT (Ser473) (1:1000, Cat#9271S), AKT (1:3000, Cat#9272S) and PTEN (1:1000, Cat#9552) were purchased from Cell Signaling Technology. Other primary antibodies used here including DLX1 (1:1000, Cat#PA5-28899, Thermo Fisher Science) and β-actin (1:10000, Cat#NB600-501, Novus). The complete photos of western blotting on these proteins were presented in the Additional file 1: Figure S4.

### Chromatin Immunoprecipitation (ChIP)

We used the EZ-Magna ChIP^™^ A/G Chromatin Immunoprecipitation kit (Cat#17–10086, Millipore) to perform ChIP experiments as the standard protocols. Briefly, in vivo cross linking was conducted via adding 1% formaldehyde to the cells (1 × 10^6^ per ChIP) for 10 min. Sonication (2*10 bursts of 30 s ON/OFF at high-level output in the sonicator (XL2015, Misonix)) of the cross-linked chromatin was performed to generate  < 500 bp DNA fragments with confirmation via running electrophoresis on the agarose gel stained with DNA VIEW (Cat#TT-DNA01, TOOLS). 1% of chromatin supernatant was collected as the input. Then, antibodies with ChIPAb + H3K27me3 (Cat#17–622, Millipore), ChIPAb + EZH2 (Cat#17–662, Millipore) or mouse IgG (Cat#12-371B, Millipore) were used to perform immunoprecipitation. For binding the antibody/antigen/DNA complex, magnetic protein A/G Beads (Cat# CS204457, Millipore) were applied. Then, ChIP samples were going through washing, reversal of cross-linking, and ChIP DNA isolation. The eluted ChIP DNA was used as the template for real-time qPCR using SYBR Green. To assess the occupancy of H3K27me3 and EZH2 on the *DLX1* gene, the primers target on *DLX1* promoter region were designed as listed in Additional file 1: Table S2.

### Immunohistochemistry staining of normal thyroid and papillary thyroid cancer tissues

The paraffin embedded sections of normal thyroid tissues (n = 22) and papillary thyroid cancer tissues with different stages (n = 52 with stage I, n = 20 with stage II, n = 15 with stage III and n = 3 with stage IVA) were purchased from US Biomax, Inc (Cat#TH8010a, Cat#TH208, Cat#TH961). Immunohistochemistry (IHC) staining of DLX1 was performed as the standard protocol including deparaffinized, rehydrating, antigen retrieval, immunohistochemical staining using the antibody against DLX1 (Cat#PA5-28899, Thermo Fisher Science), dehydrating and stabilizing with mounting medium and viewing the staining under the microscope. The staining intensity from 0 to 3 was scored, with 3 referred as the section with maximum intensity, while 0 indicated negative. The percentage of staining was estimated, and 100% staining was scored as 1. The IHC scores were calculated by staining intensity*staining percentage and assessed by an independent pathologist.

### Statistical analysis

Graphpad Prism 6.0 and SPSS version 22 were used for data analysis. The details of statistical methods were described in the figure legends.

## Supplementary Information

**Additional file 1: Figure S1. **Comparison of *HOTAIR* expression among PTCs with different genetic background. **Figure S2.** Overexpression of *HOTAIR* in the low *HOTAIR*-expressed K1 cell enhanced proliferation, colony formation and shifted the cell cycle toward G2/M phase. **Figure S3.** *DLX1* expression presented corresponding changes after *HOTAIR *modulation and had an endogenously negative correlation with *HOTAIR* levels in PTC cell lines*. Table S1.* Top 5 genes presented significantly negative correlation with *HOTAIR* expression in PTC tissues of TCGA data. **Table S2. **Used primers sequences. **Figure S4.** The corresponding changes of protein levels after *HOTAIR* modulation in MDA-T32 and MDA-T41 cell lines*. (A)* Protein markers of different cell phases, PTEN and p-AKT/AKT ratio were blotted on Scramble/*si-HOTAIR* cells *(B)* Protein markers of different cell phases, PTEN and p-AKT/AKT ratio were blotted on Control/*HOTAIR-OE* cells (*C)* DLX1 were blotted on Scramble/*si-HOTAIR *and Control/*HOTAIR-OE* cells.

## Data Availability

The datasets and materials used in this study are available from the corresponding author on reasonable request.

## Abbreviations

PTC: Papillary thyroid cancer
HOTAIR: HOX transcript antisense intergenic RNA
TCGA: The Cancer Genome Atlas
IHC: Immunohistochemical
TSH: Thyroid-stimulating hormone
lncRNA: Long non-coding RNA
PRC2: Polycomb repressive complex 2
LSD1: Lysine-specific histone demethylase 1
EZH2: Enhancer of zeste homolog 2
ER: Estrogen receptor
SNP: Single nucleotide polymorphism
HIF: Hypoxia-inducible factor
GTEx: Genotype-tissue expression
