# Supplementary material for: LncRNA HOTAIR impairs the prognosis of papillary thyroid cancer via regulating cellular malignancy and epigenetically suppressing DLX1
Source: Cancer Cell Int. 2022 Dec 9;22:396. doi: 10.1186/s12935-022-02817-2 (PMC9733112; doi:10.1186/s12935-022-02817-2)
Supplement: Supplementary file 1 — Additional file 1: Figure S1. Comparison of HOTAIR expression among PTCs with different genetic background. Figure S2. Overexpression of HOTAIR in the low HOTAIR-expressed K1 cell enhanced proliferation, colony formation and shifted the cell cycle toward G2/M phase. Figure S3. DLX1 expression presented corresponding changes after HOTAIR modulation and had an endogenously negative correlation with HOTAIR levels in PTC cell lines. Table S1. Top 5 genes presented significantly negative correlation with HOTAIR expression in PTC tissues of TCGA data. Table S2. Used primers sequences. Figure S4. The corresponding changes of protein levels after HOTAIR modulation in MDA-T32 and MDA-T41 cell lines. (A) Protein markers of different cell phases, PTEN and p-AKT/AKT ratio were blotted on Scramble/si-HOTAIR cells (B) Protein markers of different cell phases, PTEN and p-AKT/AKT ratio were blotted on Control/HOTAIR-OE cells (C) DLX1 were blotted on Scramble/si-HOTAIR and Control/HOTAIR-OE cells. [file 12935_2022_2817_MOESM1_ESM.docx]

**
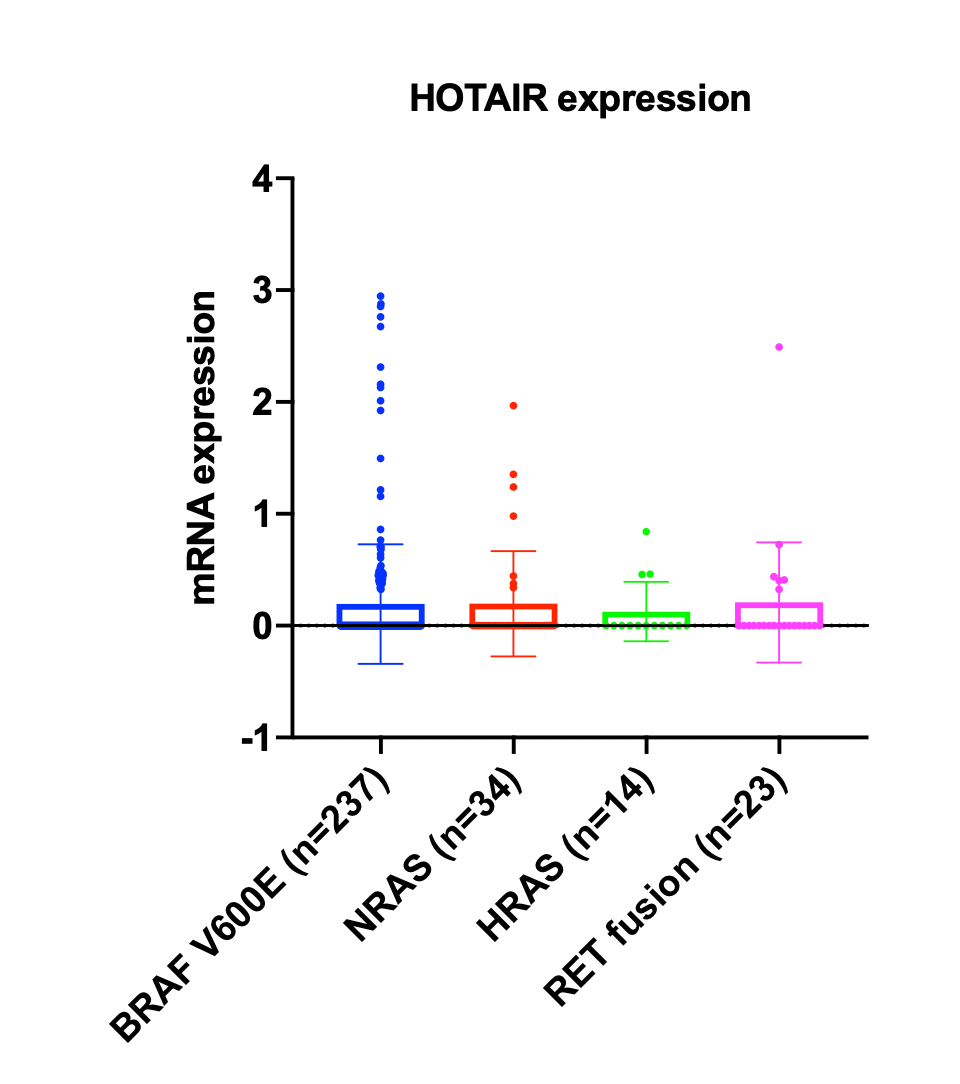
**

**Supplementary Figure 1. Comparison of *HOTAIR* expression among PTCs with different genetic background.**

Using TCGA database, we compare the *HOTAIR* levels among PTCs with different genetic background including *BRAF^V600E^*, *NRAS*, *HRAS* mutations and *RET* fusion. Data are presented as means ± SD. Statistical significance was assessed using one-way ANOVA. *p<0.05; **p<0.01; ***p<0.001.

**



**

**B**

**D**

**C**

**A**

**
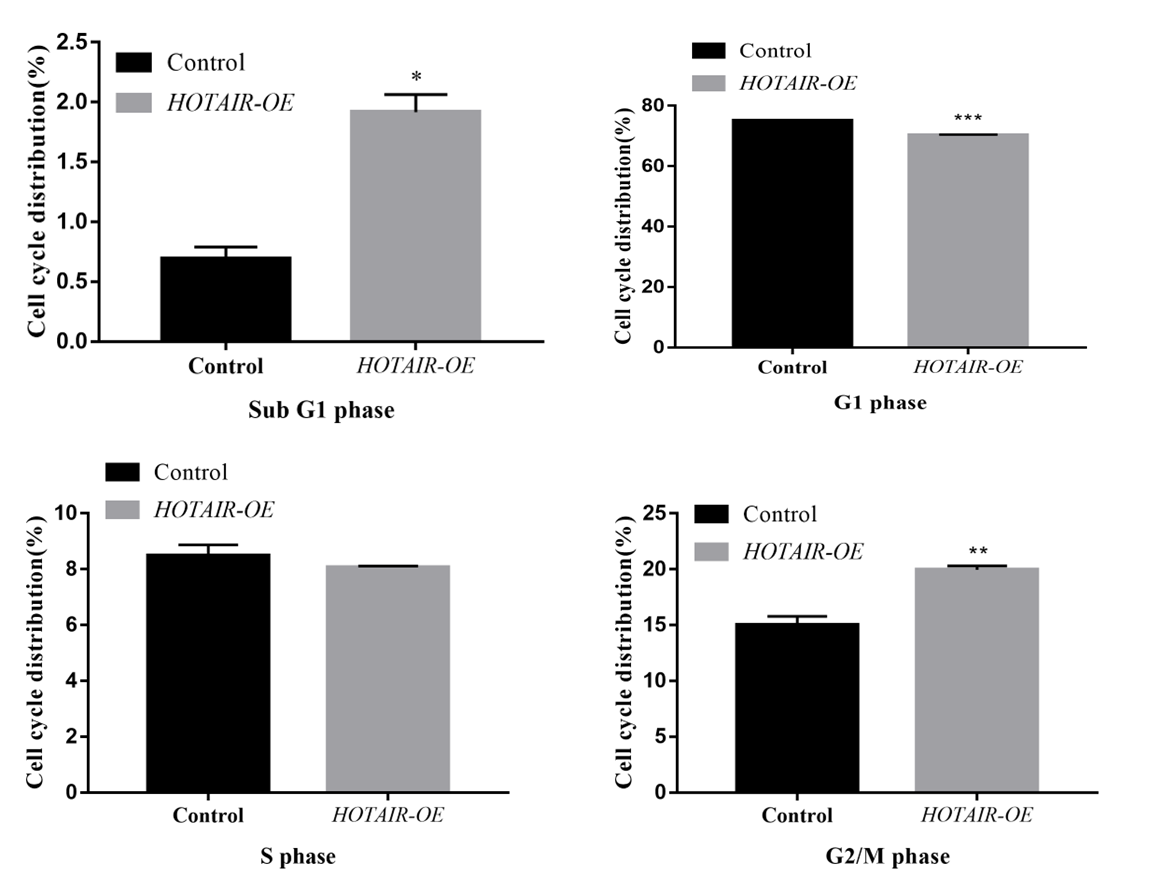

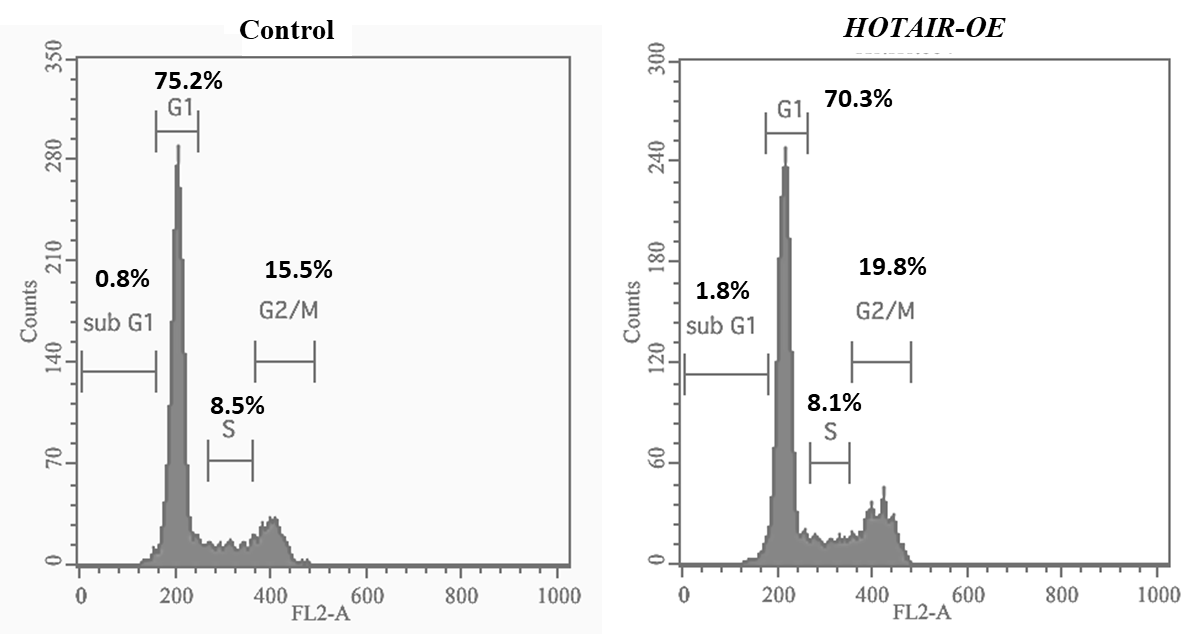
**

**K1**


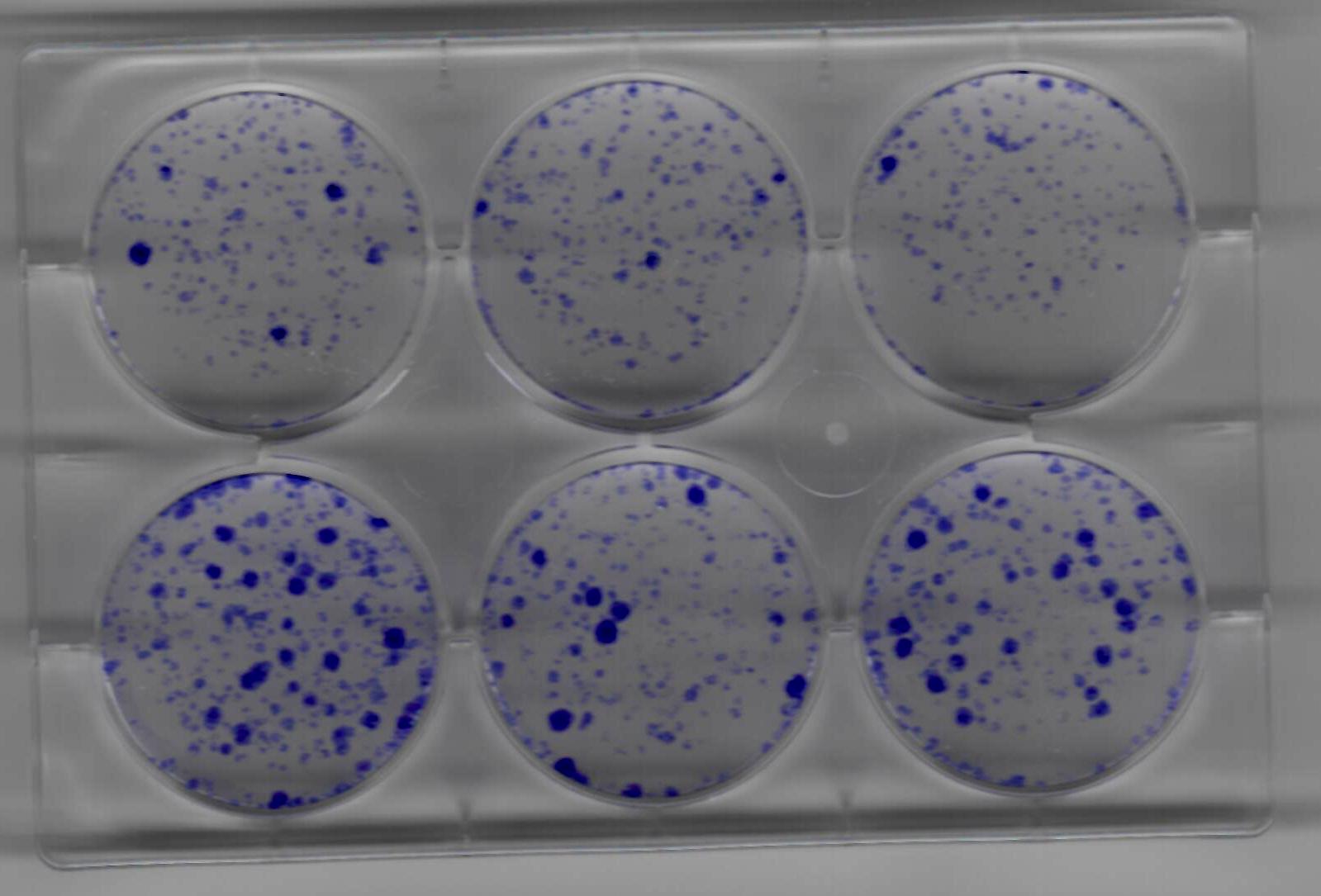

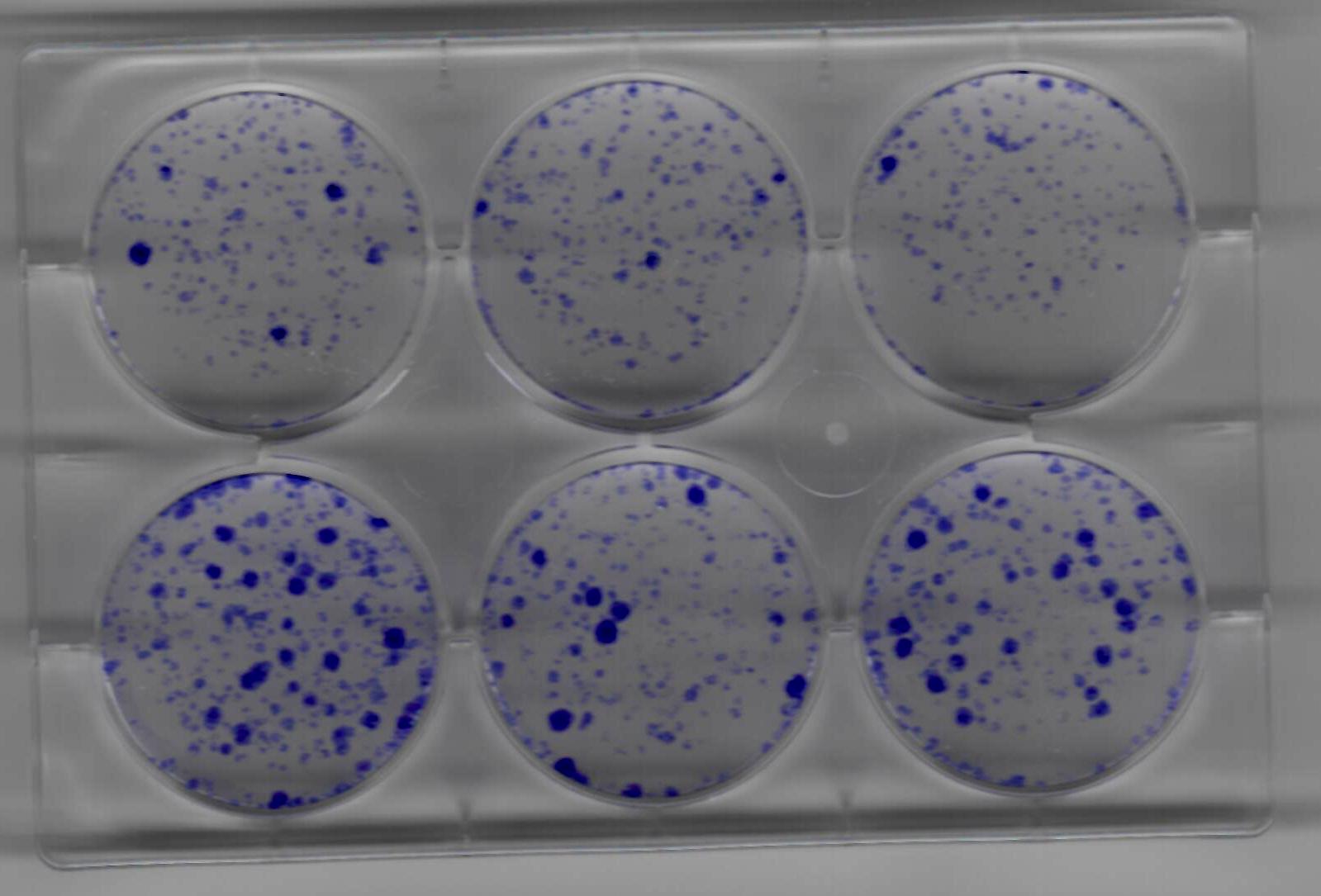


**Control**

***HOTAIR-OE***


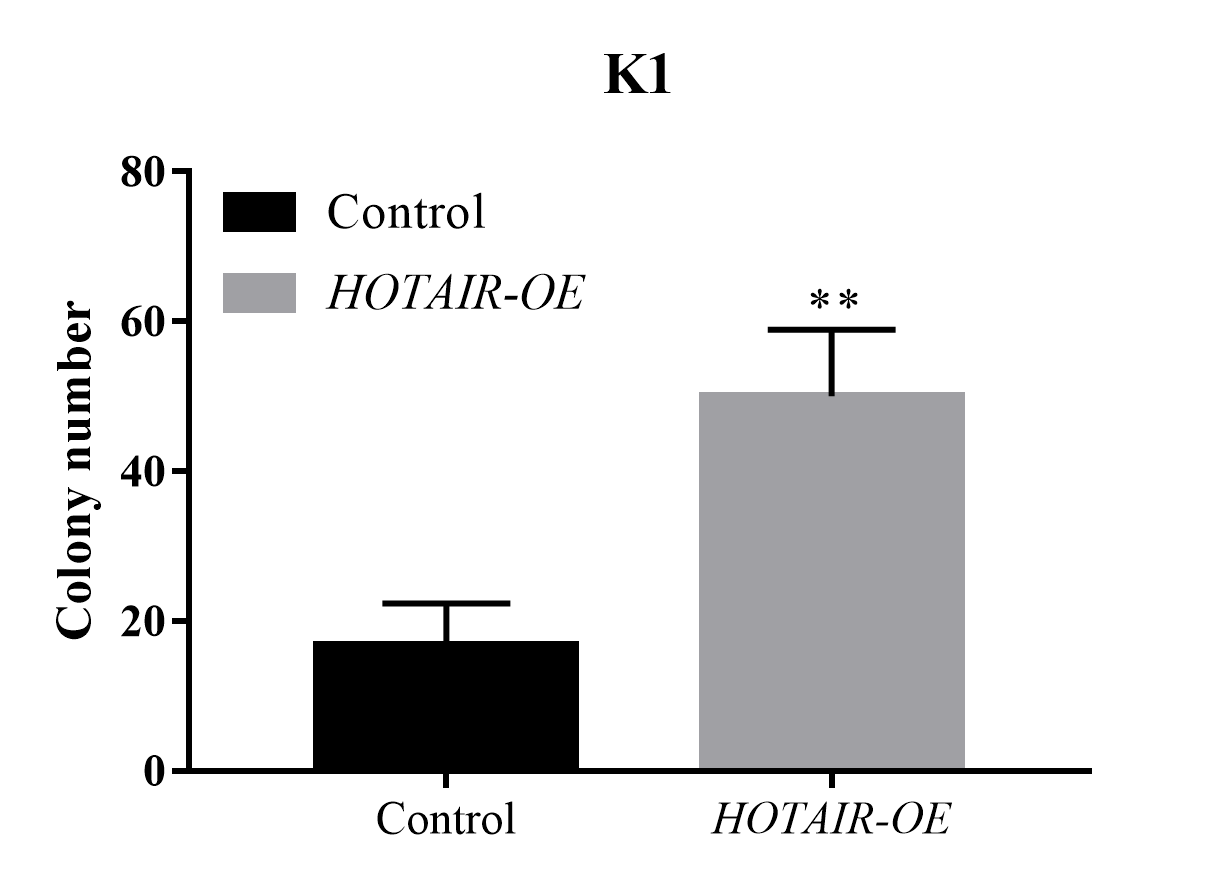


**Supplementary Figure 2. Overexpression of *HOTAIR* in the low *HOTAIR*-expressed K1 cell enhanced proliferation, colony formation and shifted the cell cycle toward G2/M phase.**

***(A)*** Constitutive *HOTAIR* overexpression in the K1 cell line (*HOTAIR-OE* cell) was generated via lentiviral transduction and empty vector was utilized to generate the Control cell (n=3). ***(B, C)*** Cell proliferation rate of Control and *HOTAIR-OE* cells was assessed by CCK-8 and colony formation assay (n=3). ***(D)*** Flow cytometry was performed among Control and *HOTAIR-OE* cells to evaluate *HOTAIR* overexpression-mediated effects on cell cycle distribution (n=3). Data are presented as means ± SD. Statistical significance was assessed using independent Student T-test. *p<0.05; **p<0.01; ***p<0.001.





**Supplementary Figure 3. *DLX1* expression presented corresponding changes after *HOTAIR* modulation and had an endogenously negative correlation with *HOTAIR* levels in PTC cell lines*. (A)*** Expression of *MAB21L2*, *BRINP3*, *HAND2-AS1*, *HAND2* and *DLX1* was measured with real-time qPCR in MDA-T32 *si-HOTAIR* and Scramble cells. ***(B)*** Expression of *MAB21L2*, *BRINP3*, *HAND2-AS1*, *HAND2* and *DLX1* was measured with real-time qPCR in MDA-T41 *HOTAIR-OE* and Control cells. Data presented as means ± SD. Statistical significance was assessed using independent Student T-test, **p*<0.05; ***p*<0.01; ****p*<0.001.

**B**

**A**

**Supplementary Table 1. Top 5 genes presented significantly negative correlation with *HOTAIR* expression in PTC tissues of TCGA data.**

| **Gene name** | **Spearman's correlation** | ***p*-Value** |
| --- | --- | --- |
| *MAB21L2* | -0.320 | 6.48E-13 |
| *HAND2-AS1* | -0.275 | 8.20E-10 |
| *BRINP3* | -0.270 | 1.73E-09 |
| *HAND2* | -0.260 | 7.07E-09 |
| *DLX1* | -0.242 | 7.61E-08 |

**Supplementary Table 2. Used primers sequences.**

| **Gene name** | **Direction** | **Sequences** |
| --- | --- | --- |
| *GAPDH* | Forward | CCACATCGCTCAGACACCAT |
|  | Reverse | TGACCAGGCGCCCAATA |
| *HOTAIR* | Forward | GGTAGAAAAAGCAACCACGAAGC |
|  | Reverse | ACATAAACCTCTGTCTGTGAGTGCC |
| *DLX1* | Forward | CATCAGTTCGGTGCAGTCCTAC |
|  | Reverse | CCTTGCCATTGAAGCGCACTTC |
| *DLX1* promoter | Forward | GATCATTTCCCCGTTCCCGT |
|  | Reverse | CCAATACAGCCAGTCCCTCG |


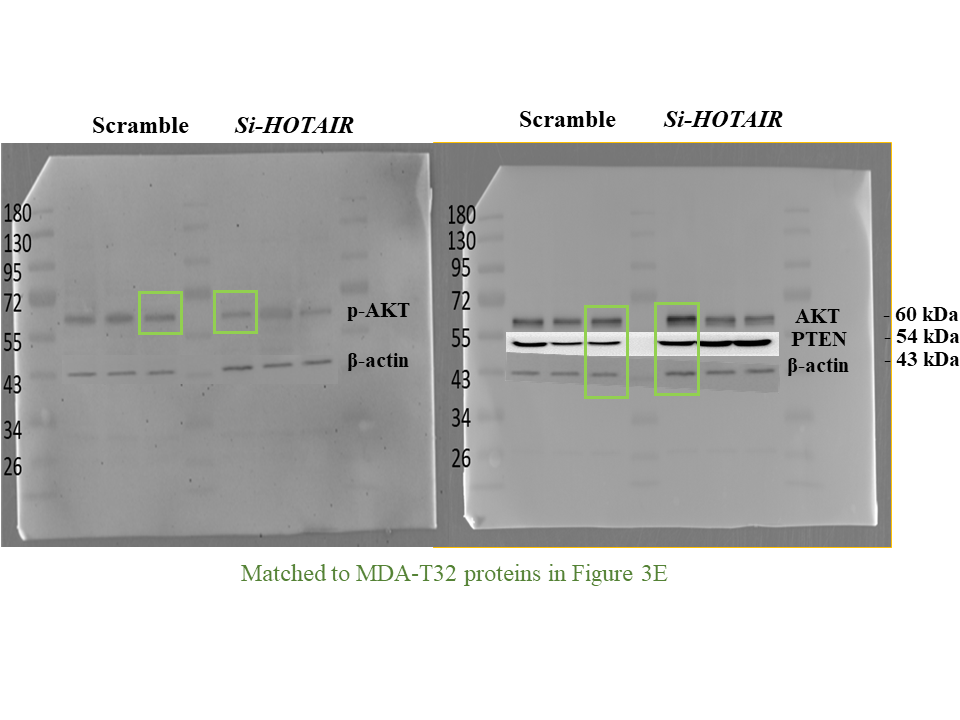

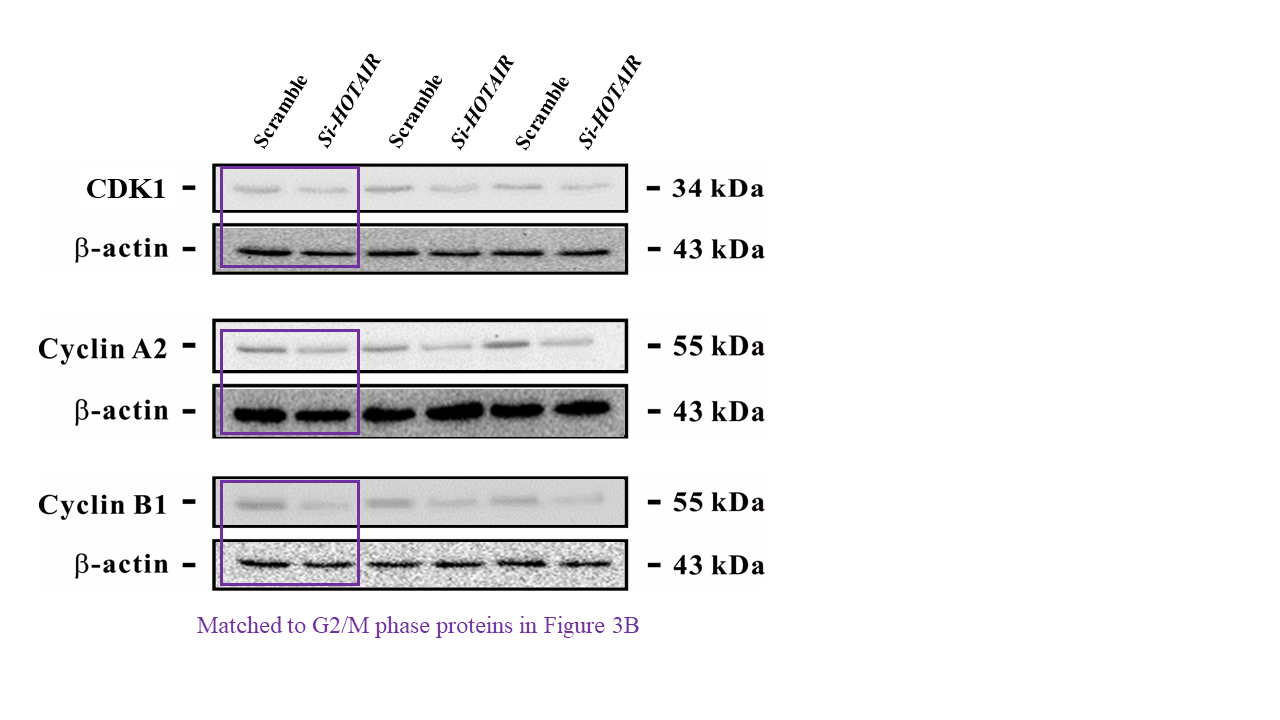

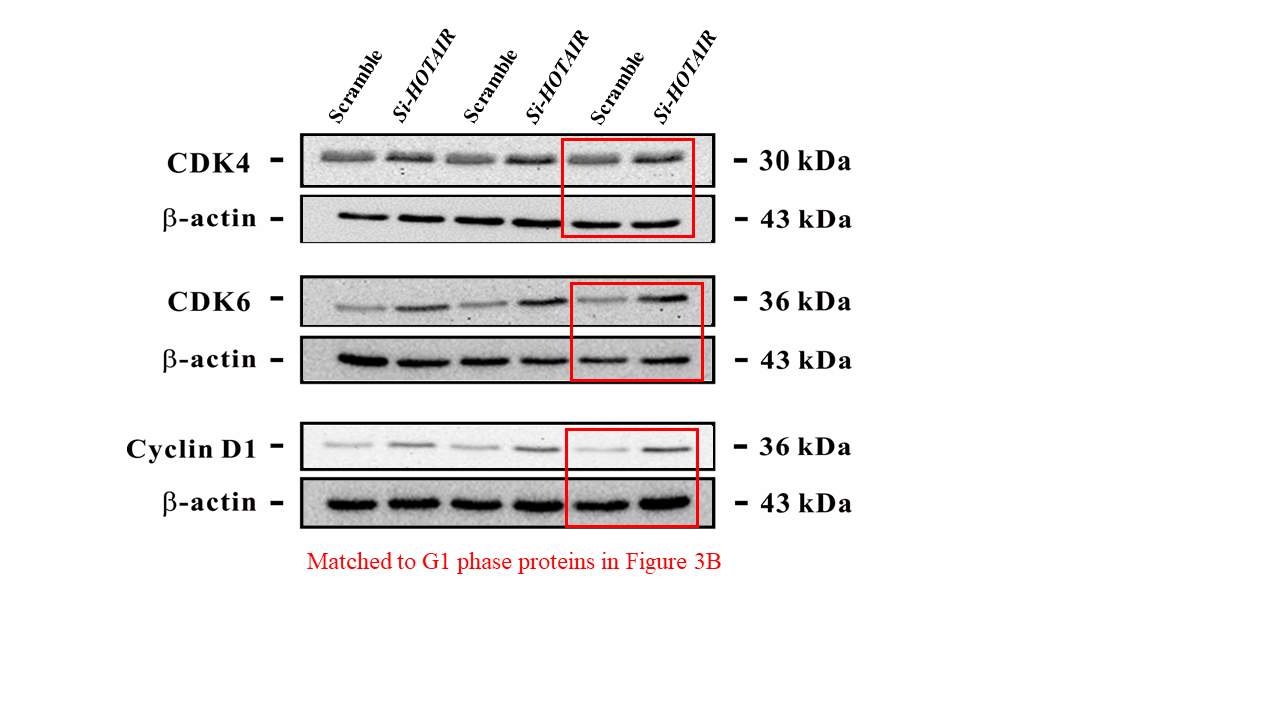

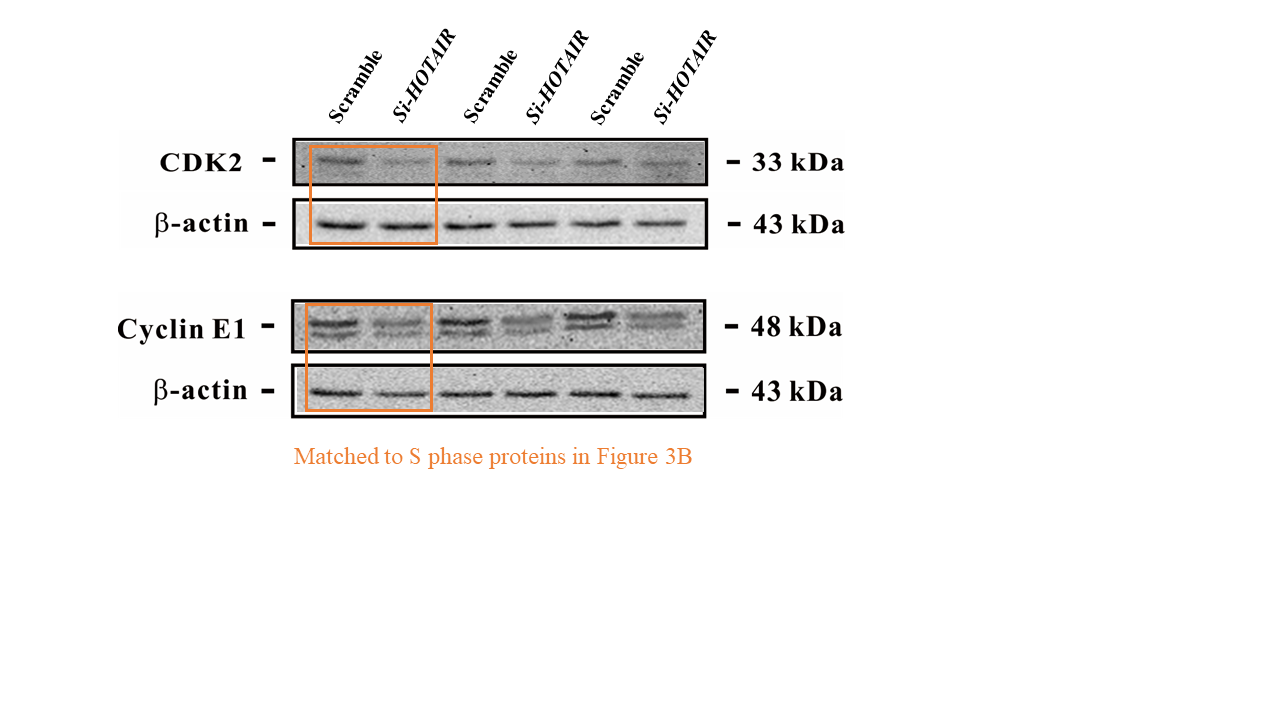


**MDA-T32**

**B**

**A**


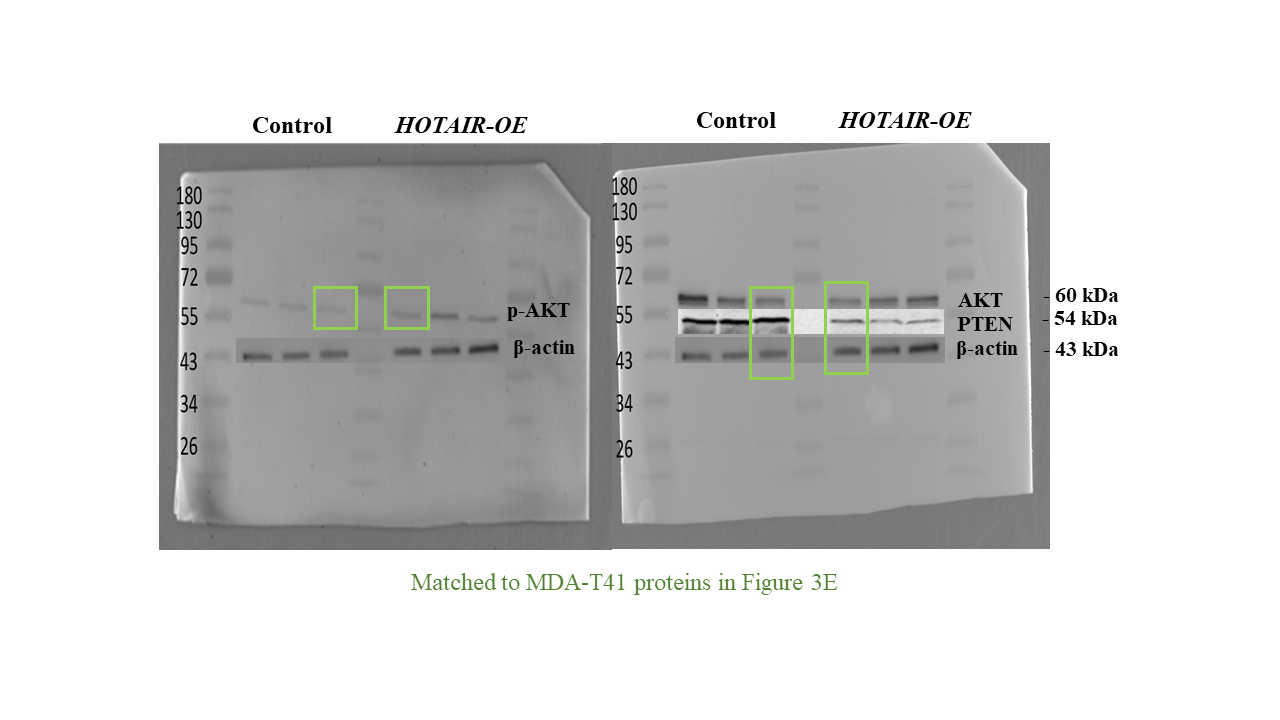

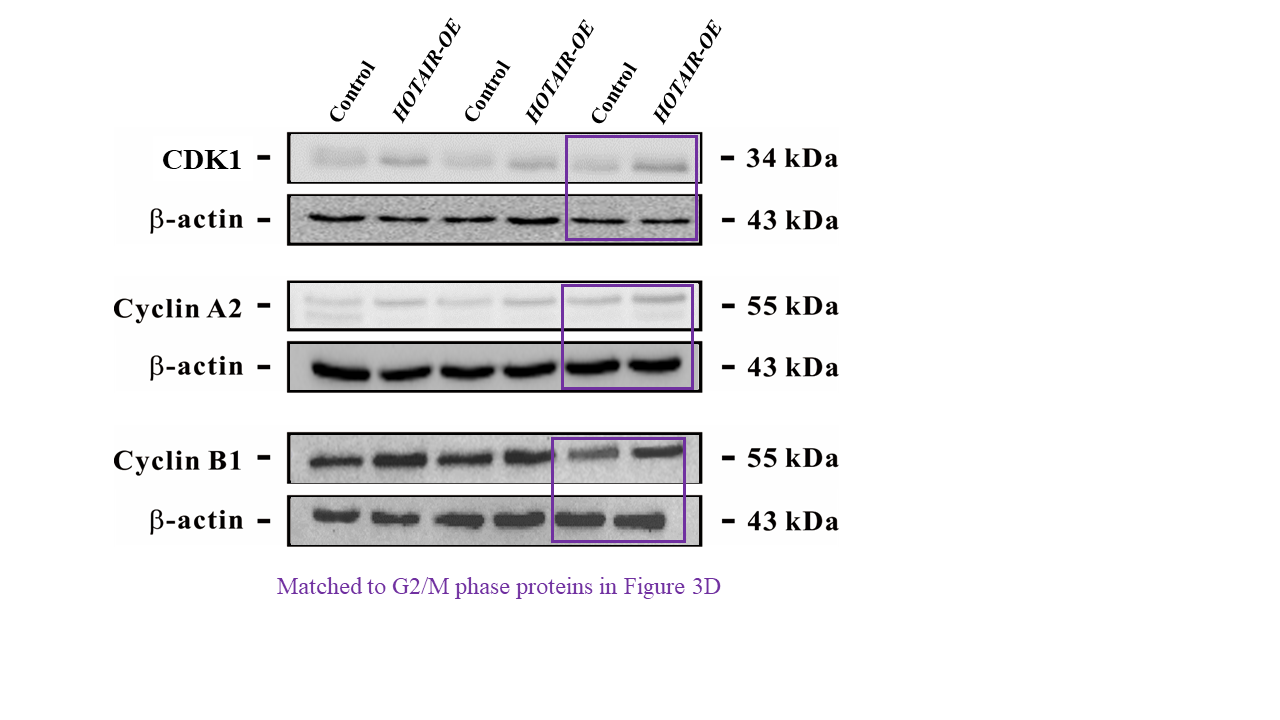

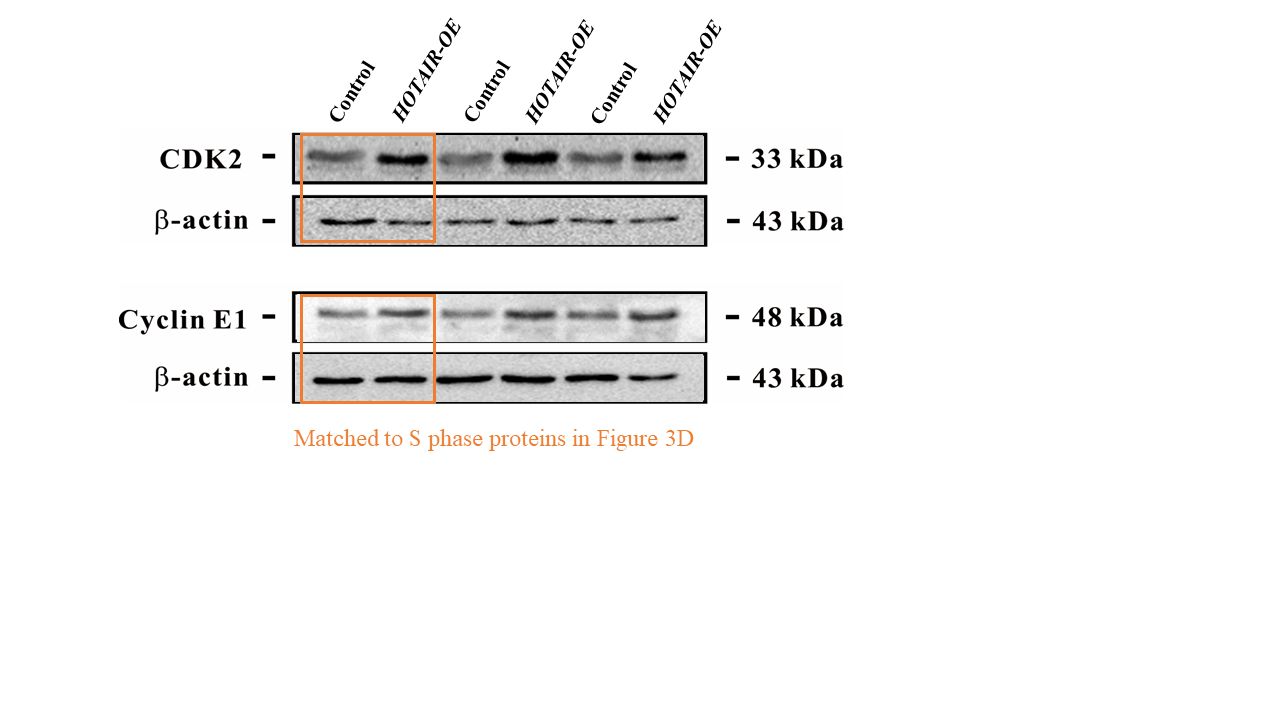

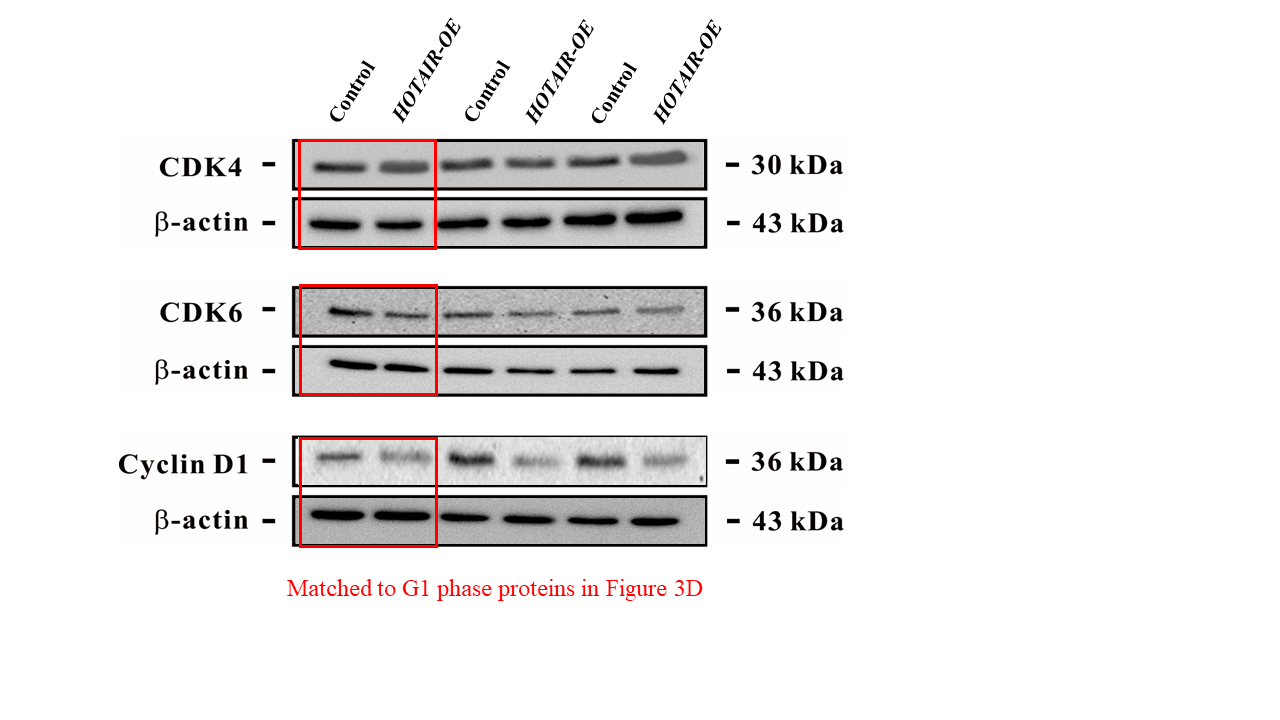


**MDA-T41**

**MDA-T32**

**MDA-T41**


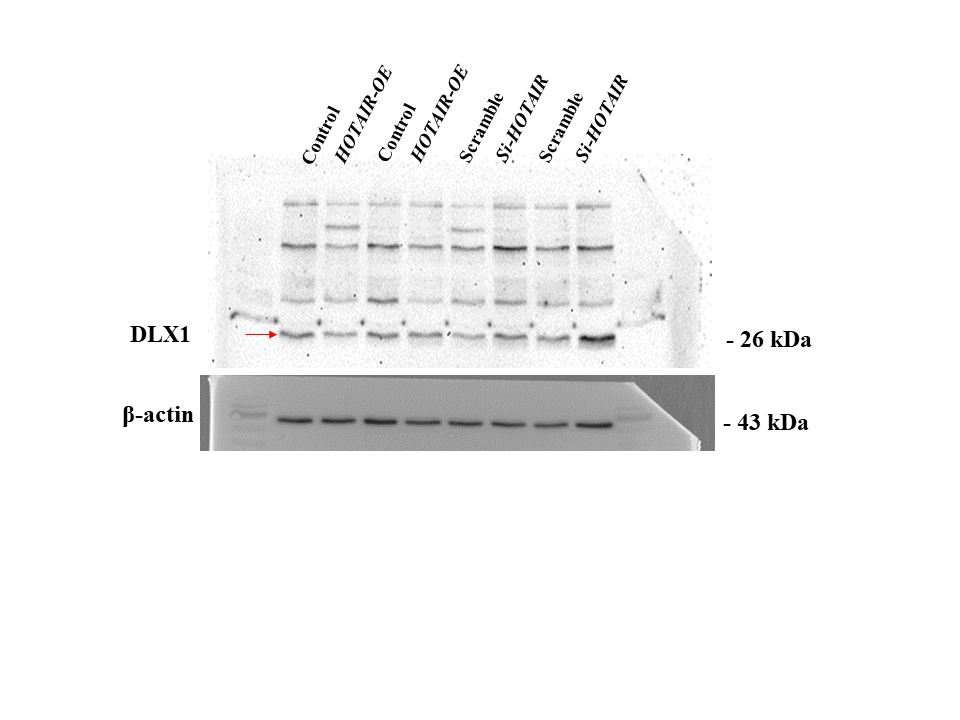


**C**

**Supplementary Figure 4. The corresponding changes of protein levels after *HOTAIR* modulation in MDA-T32 and MDA-T41 cell lines*. (A)*** Protein markers of different cell phases, PTEN and p-AKT/AKT ratio were blotted on Scramble/*si-HOTAIR* cells ***(B)*** Protein markers of different cell phases, PTEN and p-AKT/AKT ratio were blotted on Control/*HOTAIR-OE* cells (***C)*** DLX1 were blotted on Scramble/*si-HOTAIR* and Control/*HOTAIR-OE* cells.
